# Amplatzer vascular plug combined with coil and sclerosing agent for the treatment of ovarian varicocele: A case report

**DOI:** 10.1097/MD.0000000000043895

**Published:** 2025-08-15

**Authors:** Shuxiong Ge, Hui Wang, Zhongyou Xu

**Affiliations:** a Department of Vascular Surgery, People’s Hospital Affiliated to Ningbo University, Ningbo, Zhejiang Province, China.

**Keywords:** amplatzer vascular plug, endovascular interventional embolization therapy, Ovarian varicocele

## Abstract

**Rationale:**

Ovarian varicocele is a rare yet long-lasting disease in female. Endovascular interventional embolization therapy is the recommended treatment for ovarian varicocele; however, existing embolic materials are prone to causing reocclusion and recurrence of ovarian varicose veins. This study aims to assess the efficacy of vascular plugs in embolizing ovarian varicoceles, thereby providing a novel treatment option for ovarian varicose veins.

**Patient concerns:**

A 42-year-old middle-aged woman was admitted to the vascular surgery department due to persistent lower abdominal distension and pain for half a year.

**Diagnoses:**

Preoperative full abdominal enhanced CT revealed bilateral ovarian varicocele, while abdominal vascular ultrasonography showed no evidence of nutcracker syndrome.

**Interventions:**

Interventional strategy: injection of sclerosing agent into the distal branch vessels and placement of uncontrolled coils, and placement of type II vascular plug in the proximal main trunk vessels.

**Outcomes:**

The vascular embolization procedure was successfully performed. Intraoperative angiography confirmed the absence of blood reflux in the ovarian vein, and follow-up enhanced abdominal CT scans at 3 months post-operation revealed no evidence of recanalization in the ovarian vein. No adverse events were reported during the operation, and the radiation exposure time was minimal. The absence of complications during the perioperative period indicates that this method is safe. Furthermore, there was a significant improvement in postoperative abdominal pain.

**Lessons:**

This innovative method offers a safe and efficacious alternative therapeutic option for patients suffering from ovarian varicose vein.

## 1. Introduction

Chronic pelvic pain is a prevalent chronic condition among women of childbearing age, with reported prevalence rates ranging from 5.6% to 30.9%.^[1]^ Pelvic congestion syndrome, also referred to as pelvic venous insufficiency, is recognized as one of the potential causes of chronic pelvic pain. It is characterized by pathological conditions such as retrograde flow in the ovarian vein, venous dilation, and parametrial varices.^[2]^ Ovarian varicocele represents one of several etiological factors contributing to pelvic venous insufficiency and is often underdiagnosed in clinical practice. Given that pelvic pain significantly impacts patients’ physical health and quality of life, it is crucial to enhance our understanding of this condition and identify modifiable risk factors. With advancements in diagnostic and therapeutic techniques, an increasing number of cases of ovarian varicocele have been identified. Effective management and treatment of ovarian varicocele have become imperative. Minimally invasive treatments for ovarian varicocele are now the predominant approach, with relatively well-established efficacy. Various interventional embolization methods are available for treating ovarian varicose vein, including coil, sclerosing agent, and gelatin sponge, all of which can effectively achieve embolization and treatment.^[3]^ However, some studies suggest that mechanical material embolization of ovarian vein may lead to recurrence, and there is insufficient evidence to determine the optimal embolization material for ovarian varicocele. The amplatzer vascular plug (AVP), a novel embolic material, is composed of tightly woven multiple layers of nickel–titanium alloy wires without internal fillers, offering advantages such as excellent compressibility and navigability.^[4]^ This case report describes the embolization treatment of ovarian varicocele using a combination of amplatzer vascular plug, coil, and sclerosing agent.

## 2. Case report

**Figure 1.**
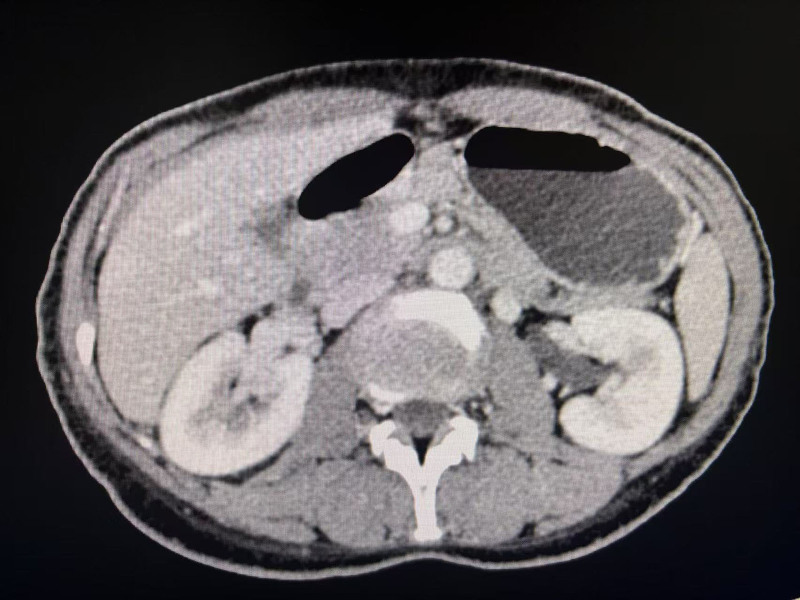
The full abdominal enhanced CT scan reveals ovarian varicosity. The asterisk denotes the presence of varicose ovarian vein.

A 42-year-old woman was admitted to the vascular surgery department in December 2024 with a 6-month history of lower abdominal distension and pain. The patient had a medical history of diabetes, which was managed with oral hypoglycemic agents, and no history of hypertension. On physical examination, there were no obvious signs of ovarian varicocele in the abdomen; the abdomen was soft without tenderness or rebound pain, and no varicose veins were noted in the lower extremities. An enhanced CT scan of the abdomen revealed bilateral ovarian varicosities (Fig. 1). Following the preoperative departmental discussion, we determined that the optimal treatment for the patient’s ovarian varicose vein would be a combination of interventional vascular embolization using AVP, coil and sclerosing agent therapy.

**Figure 2.**
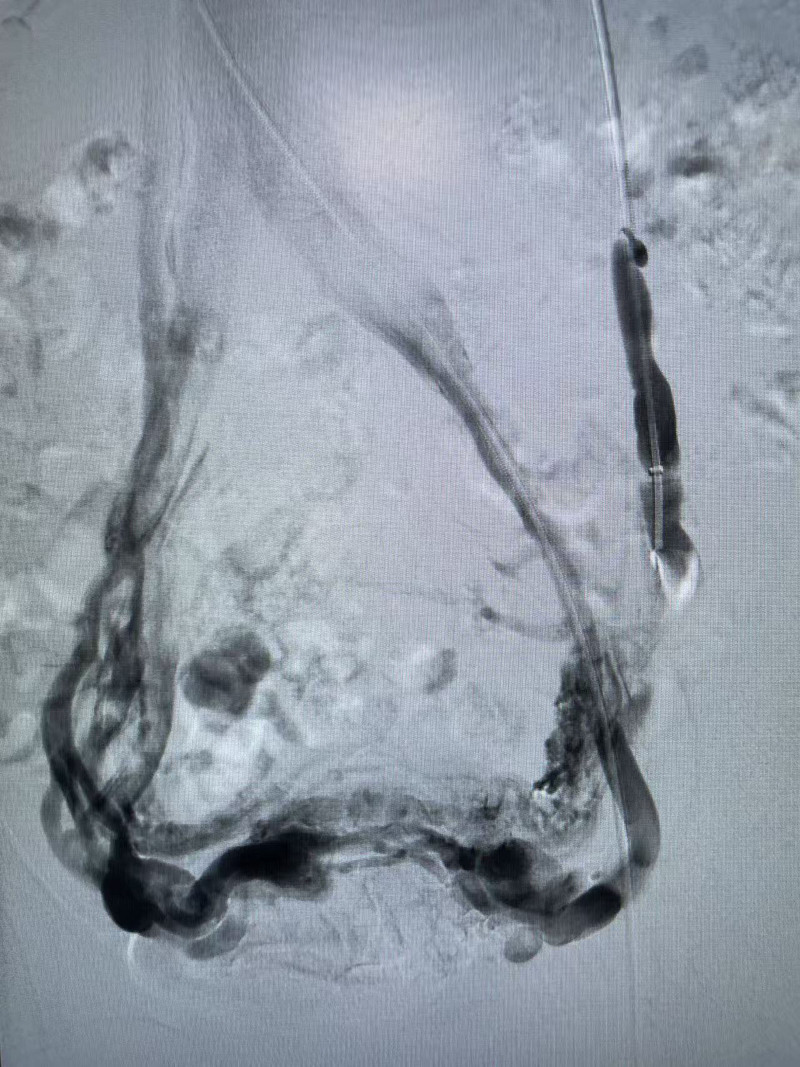
Intraoperative DSA angiography demonstrated significant dilation of the left ovarian vein, retrograde blood flow, pelvic varicosities, and extensive collateral circulation.

**Figure 3.**
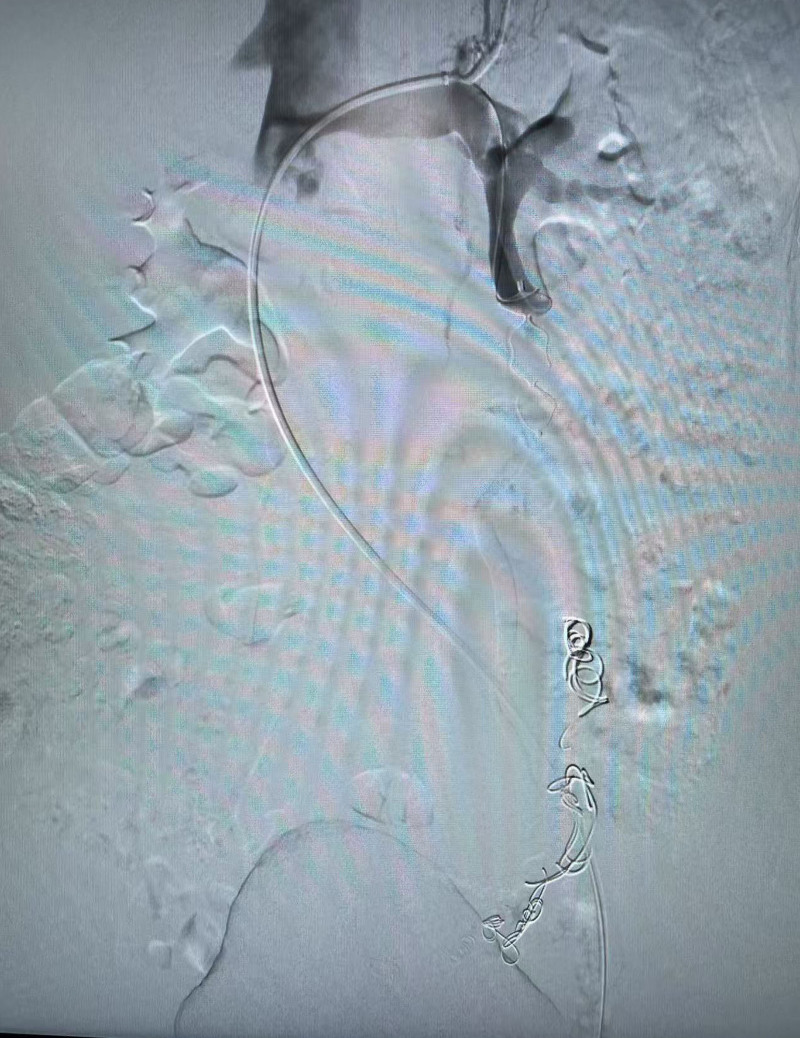
The amplatzer vascular plug and coil were used to achieve complete embolization of the left ovarian vein, with no significant backflow observed during the follow-up period.

Conventional disinfection and draping were performed. Following successful local infiltration anesthesia in the left groin, a 6F puncture needle was introduced through the left femoral vein, and subsequently, a 6F long sheath was exchanged. Under fluoroscopic guidance, the catheter, in conjunction with the guidewire, was advanced into the left renal vein and the left ovarian vein. Angiography revealed that the left ovarian vein was markedly dilated, exhibiting retrograde blood flow, multiple varicosities, and extensive collateral circulation, with blood refluxing via the right iliac vein (Fig. 2). Following the systemic administration of 5000 units of heparin, the distal branches of the ovarian vein were embolized using 3 nondetachable coils measuring 8 mm in diameter and 3 measuring 15 mm in diameter. Subsequently, approximately 8 mL of a 3% polidocanol foam solution was injected into the left ovarian vein. Upon completion of distal embolization, the microcatheter was retracted, and a 16 mm AVP (Abbott Cardiovascular, Chicago, IL) was deployed. Reangiography confirmed successful embolization of the left ovarian vein, with no contrast agent leakage observed (Fig. 3). Additionally, the tortuous veins in the pelvic cavity have significantly decreased. Pressure measurements taken from the middle segment of the left renal vein and the inferior vena cava using a pressure catheter revealed no significant differences. Based on these findings, it was determined that placement of a stent in the left renal vein was unnecessary. The procedure concluded with device withdrawal and application of a pressure bandage to the left groin. After a 3-month follow-up post-surgery, the patient’s abdominal pain symptoms showed significant improvement. The enhanced abdominal CT scan revealed that the ovarian vein had not recanalization, and the tortuosity of the pelvic vein had been alleviated. This study was conducted with the approval of the hospital ethics committee and after obtaining informed consent from the patients. Additionally, written consent for the publication of this case was obtained from the patient. The datasets generated during and analyzed during the current study are available from the corresponding author on reasonable request.

## 3. Discussion

Chronic pelvic pain is a significant health issue affecting women. Historically, due to limitations in diagnostic and therapeutic capabilities, ovarian varicose vein was often overlooked as a potential cause. In recent years, with growing awareness of women’s health issues, ovarian varicose vein has garnered increasing attention from the medical community. The primary goal of treating ovarian varicose vein is to achieve effective pain relief. Intracavitary embolization therapy has the advantages of small trauma, high safety and high technical success rate, and has become the preferred treatment option for ovarian varicose vein. The continuous innovation of embolization materials has driven the rapid progress of embolization technology. However, the embolization effect and complications need to be further considered. The feature of this case is that it provides an opportunity to utilize new embolization materials for the treatment of ovarian varicose vein.

AVP is self-expanding nitinol devices available in 4 types. Among these, the AVP type 2 (AVP-2) and AVP type 4 (AVP-4) are the most commonly utilized models for embolization procedures.^[5]^ The AVP-4 is particularly suited for tortuous and small-caliber vessels, whereas the AVP-2 is more appropriate for large-caliber vessels and a variety of landing zones.^[6]^ Vascular plug has been documented in applications such as splenic artery, internal iliac vein, arteriovenous fistula, spermatic vein, and stent leak.^[5]^ However, their use in ovarian vein has been infrequently reported. Literature on ovarian vein embolization is limited, and the recurrence and recanalization rates remain unclear. Regarding varicocele, several scholars conducted a systematic review of the safety and efficacy of various embolic materials used in its treatment. They analyzed data from 30 clinical studies encompassing a total of 3505 cases and found that the average recurrence rate was highest for sclerosing agents (11.03%), while it was lower for glue (4.2%).^[7]^ The recurrence rates for coil alone or in combination with sclerosing agent fell between these 2, at 9.1% and 8.44%, respectively. It is evident that the choice of embolic material remains critically important for preventing recurrence. Moreover, the recurrence of ovarian varicose vein may be associated with the opening of distal collateral vessels and the recanalization of the primary blood vessels. Antonio Basile reported a successful case where the combination of sclerosing agent and vascular plug effectively controlled the symptoms.^[8]^ The combined application of multiple embolization techniques is a crucial factor in reducing the recurrence of embolism. In this case, distal molecular veins were embolized using a combination of sclerosing agent and coil, while the main trunk vein was occluded with vascular plug, thereby minimizing the risk of recanalization. Follow-up examinations 3 months post-operation demonstrated excellent embolization outcomes. In this study, we examined a limited number of cases to assess the efficacy of AVP in the treatment of varicocele. However, more extensive data is required to substantiate the effectiveness of this intervention. Furthermore, the 3-month follow-up period we employed is relatively short; therefore, longer follow-up durations are necessary to validate the long-term effectiveness of vascular embolization. The successful implementation of this approach not only paves the way for the broader application of AVP across various diseases but also introduces new material for the treatment of ovarian varicose vein.

The advantages of AVP in the treatment of ovarian vein are as follows: (1) Excellent conformity: Upon deployment, the plug can adapt to irregularly shaped blood vessels, ensuring optimal positioning and function. (2) Retrievability: The plug can be pre-released into the vessel for evaluation; if the initial placement is unsatisfactory, it can be retrieved into the sheath and repositioned multiple times until the ideal location is achieved before detaching from the delivery system. (3) Thrombogenicity: The dense mesh structure on the surface of the vascular plug provides a stable scaffold for platelet adhesion and aggregation, promoting effective thrombus formation. (4) High cost-effectiveness: A single vascular plug offers functionality equivalent to multiple controllable coils, reducing overall material costs. (5) Reduced complications: The design minimizes migration risk, contributing to enhanced patient safety.

## 4. Conclusion

In conclusion, AVP for the treatment of ovarian varicocele demonstrates high safety and efficacy, characterized by a high success rate, straightforward surgical procedures, and minimal complications. This technique is highly recommended for wider adoption.

## Author contributions

**Software:** Shuxiong Ge.

**Supervision:** Zhongyou Xu.

**Writing – original draft:** Shuxiong Ge.

**Writing – review & editing:** Hui Wang, Zhongyou Xu.

## References

[1] Ayorinde AA Bhattacharya S Druce KL Jones GT Macfarlane GJ. Chronic pelvic pain in women of reproductive and post-reproductive age: a population-based study. Eur J Pain. 2017;21:445–55. 27634190 10.1002/ejp.938

[2] Durham JD Machan L. Pelvic congestion syndrome. Semin Intervent Radiol. 2013;30:372–80. 24436564 10.1055/s-0033-1359731 PMC3835435

[3] Ahuja RS Garg T Sudheendra D. Management of patients when superficial venous disease arises from pelvic escape points. Semin Intervent Radiol. 2021;38:226–32. 34108810 10.1055/s-0041-1729744 PMC8175109

[4] Nagatomi S Ichihashi S Yamamoto H Bolstad F Kichikawa K. Coil-in-plug technique using the amplatzer vascular plug II to occlude a portosystemic shunt. Vasc Endovascular Surg. 2022;56:121–5. 34237235 10.1177/15385744211032454

[5] Lopera JE. The amplatzer vascular plug: review of evolution and current applications. Semin Intervent Radiol. 2015;32:356–69. 26622098 10.1055/s-0035-1564810 PMC4640916

[6] Pfeifer J Gheibeh A Fries P Poryo M Rentzsch A Abdul-Khaliq H. Transcatheter embolization in congenital cardiovascular malformations—variable use of vascular plugs. Cardiovasc Ther. 2024;2024:4778469. 39742028 10.1155/2024/4778469 PMC11221997

[7] Makris GC Efthymiou E Little M. Safety and effectiveness of the different types of embolic materials for the treatment of testicular varicoceles: a systematic review. Br J Radiol. 2018;91:20170445. 29493263 10.1259/bjr.20170445 PMC6209458

[8] Basile A Marletta G Tsetis D Patti MT. The amplatzer vascular plug also for ovarian vein embolization. Cardiovasc Intervent Radiol. 2008;31:446–7. 18060455 10.1007/s00270-007-9235-y

